# Supplementary material for: Content-rich biological network constructed by mining PubMed abstracts
Source: BMC Bioinformatics. 2004 Oct 8;5:147. doi: 10.1186/1471-2105-5-147 (PMC528731; doi:10.1186/1471-2105-5-147)
Supplement: Additional File 2 — The original results of the above study (non-essential files are deleted to keep the file size under the limit set by BMC bioinformatics). [file 1471-2105-5-147-S2.bz2 › chilibotAdditionalFile2/dip30/296ID7486683E1287/html/GRB2_IRS1.html]

 


 **GRB2** and **IRS1** 
  
Found 180 abstracts in PubMed, retrieved 30.  
 

 What does Google say? 
 PDF only 
| .edu only 

---

**Interactive relationship** (e.g. stimulation, inhibition, etc)

**Stimulatory relationship**- Moreover, the GH induced association of IRS 1  [ **IRS1** ]  phosphatidylinositol 3 kinase, IRS 1  [ **IRS1** ]  growth factor receptor bound 2  **Grb2** , and Shc  **Grb2**  are increased in diabetic rats as well.  Ref: 12086960 Diabetes, 2002
- Although phosphatidylinositol 3 kinase PI3K and Grb 2  [ **GRB2** ]  interact with tyrosine phosphorylated IRS  [ **IRS1** ]  proteins, Shp 2 only binds to IRS  [ **IRS1** ]  proteins following IL 4, but not IL 9, stimulation.  Ref: 11788580 J Biol Chem, 2002
- insulin is able to induce association of insulin receptor substrate 1  [ **IRS1** ]  phosphatidylinositol 3 kinase and Shc growth factor receptor bound protein 2  [ **GRB2** ]  in this tissue, as well as phosphorylation of mitogen activated protein kinase and serine threonine kinase, demonstrating that proteins involved in early steps of insulin action are expressed in skin of intact rats and are quickly activated after insulin stimulation.  Ref: 11676840 J Invest Dermatol, 2001
**Neutral relationship**- Upon tyrosine phosphorylation, IRS 1  [ **IRS1** ]  binds to signaling molecules that express Src homology 2 SH 2 binding domains, including phosphatidylinositol 3 kinase PI 3 kinase, phosphotyrosine phosphatase SHP 2 Syp, Nck, Crk and Grb 2  [ **GRB2** ] .  Ref: 11279262 J Neurochem, 2001
- Signalling by the insulin receptor substrate IRS  [ **IRS1** ]  proteins is critically dependent on the tyrosine phosphorylation of specific binding sites that recruit Src homology 2 SH2 domain containing proteins, such as the p85 subunit of phosphoinositide 3 kinase PI 3 kinase, the tyrosine phosphatase SHP 2 and the adapter protein  **Grb2** .  Ref: 12220227 Biochem J, 2002
- With regard to the large effect on MAPK phosphorylation, we found that PTP1B overexpression led to the impaired phosphorylation of both IRS 1  [ **IRS1** ]  and Shc, resulting in a decrease in their association with  **Grb2** .  Ref: 12446583 Endocrinology, 2002

**Non-interactive relationship** (e.g. studied together, co-existance, homology, etc.)

- Data presented here provide strong evidence that IGF IR deficient brown adipocytes show an increased insulin sensitivity via IRS 1  [ **IRS1** ]  Grb 2  [ **GRB2** ]  MAPK, resulting in an increased mitogenesis in response to insulin.  Ref: 11872675 Diabetes, 2002
- The nanomolar affinity of the mAZ pY alphaMe pY N NH2 pseudo peptide for  **Grb2**  SH2 is related to new interactions with non conserved residues.The design of  **Grb2**  SH2 domain inhibitors that prevent interaction with tyrosine kinase proteins or other adaptors like Shc or  **IRS1**  should provide a means to interrupt the Ras signaling pathway.  Ref: 11827484 J Mol Biol, 2002
- We show that in Drosophila mutating the consensus binding site for the Ras pathway adaptor Drk  **Grb2**  in Chico IRS  [ **IRS1** ]  does not interfere with growth whereas mutating the binding sites of the PI 3 kinase adaptor p60 completely abrogates Chico function.  Ref: 12163412 Development, 2002
- Downstream, the association IRS 1  [ **IRS1** ]  growth factor receptor binding protein 2 Grb 2  [ **GRB2** ]  was augmented in the IGF IR brown adipocyte cell line.  Ref: 11872675 Diabetes, 2002
- The results from the overexpression of IRAS, where its amount was about the same as that of IRS  [ **IRS1** ]  4, indicated that IRAS associated directly with IRS  [ **IRS1** ]  4 and showed that the increased complexation of IRS  [ **IRS1** ]  4 with IRAS did not alter the insulin stimulated tyrosine phosphorylation of IRS  [ **IRS1** ]  4 or the association of IRS  [ **IRS1** ]  4 with phosphatidylinositol 3 kinase or  **Grb2** .  Ref: 11912194 J Biol Chem, 2002
- IRS 1  [ **IRS1** ]  association with  **Grb2**  and PI3K p85, which link IGFRI to the Ras MAPK and PI3K Akt pathways, respectively, was enhanced in both cell types in the presence of EN.  Ref: 12173038 Oncogene, 2002
- In response to IGF 1 treatment, the expression of kinases in the c Jun c Jun NH 2 terminal kinase signaling pathway MEKK1, MEK4, and JNK1 2 was unchanged, but expressions of insulin receptor substrate 1  [ **IRS1** ]  and  **Grb2**  associated binder 1 Gab1 were significantly decreased.  Ref: 12065326 Circ Res, 2002
- EGF increased the tyrosine phosphorylation of several proteins the EGF receptor, insulin receptor substrate IRS 1  [ **IRS1** ] , IRS  [ **IRS1** ]  2, and  **Grb2**  associated binder 1.  Ref: 12138086 J Biol Chem, 2002
- TGF beta1 affected IGF I stimulated insulin receptor substrate 1  [ **IRS1** ]  IRS 1  [ **IRS1** ]  tyrosine phosphorylation and its association with  **Grb2**  protein.  Ref: 12837295 Exp Cell Res, 2003
- Exposure of these cells to ghrelin caused up regulation of several insulin induced activities including tyrosine phosphorylation of insulin receptor substrate 1  [ **IRS1** ]  IRS 1  [ **IRS1** ] , association of the adapter molecule growth factor receptor bound protein 2  [ **GRB2** ]  with IRS 1  [ **IRS1** ] , mitogen activated protein kinase activity, and cell proliferation.  Ref: 11724768 J Biol Chem, 2002
- The tyrosine phosphorylation of IGF IR, IRS  [ **IRS1** ]  2, Shc, and p42 p44 MAP kinase MAPK, and the association of Grb 2  [ **GRB2** ]  with Shc, were examined by immunoprecipitations and Western blotting.  Ref: 11505033 Alcohol Clin Exp Res, 2001
- In particular, IGF IR appears to couple IRS 1  [ **IRS1** ]  preferentially to  **Grb2**  whereas, in contrast, IR appears to couple IRS 1  [ **IRS1** ]  preferentially to the p85 subunit of phosphatidyl inositol 3 kinase PI3 kinase and to Nck.  Ref: 11572799 J Endocrinol, 2001
- Treatment with 17 AAG decreased NPM ALK expression and phosphorylation, thus impairing its association with phospholipase C gamma, Src homology 2 domain containing protein Shc, growth factor receptor bound protein 2  [ **GRB2** ]   **Grb2** , and insulin receptor substrate 1  [ **IRS1** ]  IRS 1  [ **IRS1** ] .  Ref: 11888936 Cancer Res, 2002
- Conversely, TGF beta1 did not alter IGF I stimulated IRS 1  [ **IRS1** ]  associated phosphatidylinositol 3 kinase activity, IGF I induced tyrosine phosphorylation of Shc, and its binding to  **Grb2** .  Ref: 12837295 Exp Cell Res, 2003
- We observed association between BCR ABL IRS 1  [ **IRS1** ] , IRS 1  [ **IRS1** ]  phosphoinositide 3 kinase PI3 kinase, and IRS 1  [ **IRS1** ]   **Grb2**  in the K562 cell line.  Ref: 12560071 FEBS Lett, 2003
